# Renal biopsy in patients with type 2 diabetes mellitus: indications and nature of the lesions

**DOI:** 10.4103/0256-4947.57167

**Published:** 2009

**Authors:** Amal Abdel Ghani, Salah Al Waheeb, Ali Al Sahow, Naser Hussain

**Affiliations:** a From the Nephrology Unit, Mubarak Al Kabeer Hospital, Ministry of Health, Kuwait; b From the Department of Histopathology, Mubarak Al Kabeer Hospital, Ministry of Health, Kuwait; c From the Nephrology Unit, Jahra Hospital, Ministry of Health, Kuwait

## Abstract

**BACKGROUND AND OBJECTIVES:**

The prevalence of non diabetic renal disease (NDRD) among patients with type 2 diabetes mellitus varies widely depending on the selection criteria and the populations being studied. The aim of this study was to evaluate the renal biopsies performed on type 2 diabetic patients for suspicion of NDRD and to correlate the pathological with the clinical and laboratory findings.

**SUBJECTS AND METHODS:**

We selected and reviewed biopsies performed on type 2 diabetics for clinically suspected NDRD from January 2006 to December 2008 at a single hospital. Clinical and laboratory data were analyzed in relation to the histopathology findings. Patients were grouped into either group I with isolated DGS or group II with NDRD on top of DGS.

**RESULTS:**

Thirty-one biopsies were performed on type 2 diabetic patients; Seventeen patients (54.8%) were males. Mean age was 50.68 (11.29) years. The mean duration of diabetes was 9.33 (3.6) years. Renal biopsy showed that among the studied group 14 patients (45.2%) showed NDRD on top of DGS. Crescentic glomerulonephritis was the commonest finding seen in 3 cases (21.4% of group II cases) followed by acute tubulointerstitial nephritis and hypertensive changes each was seen in 2 cases (14.4%). Other findings included IgA nephropathy, primary focal segmental glomerulosclerosis, rhabdomyolysis, membranoproliferative glomerulonephritis each of them was seen in one case (7.1%). Group I had a significantly higher level of proteinuria 4.97 (2.08) gm/24 hrs urine than group II 2.72 (1.09) gm/24 hrs urine (*P*=.003). There was no significant difference between the two groups in age, duration of diabetes, gender, presence of hypertension, hematuria, serum creatinine or glomerular filtration rate.

**CONCLUSION:**

The present study showed that crescentic glomerulonephritis is the commonest NDRD among diabetic patients. A higher level of proteinuria was reported among those with NDRD superimposed on DGS. So, Renal biopsy should be performed in diabetics when the clinical scenario is atypical.

Diabetic nephropathy (DN) is characterized by persistent proteinuria, hypertension, and a progressive decline in renal function.^1^ About 20% to 40% of patients suffering from diabetes mellitus eventually develop diabetic renal disease.^2^ It accounts for over 40% of new cases of end-stage renal disease (ESRD) annually, and is considered the leading cause of ESRD in the USA as well as Europe and Japan.^2^ The characteristic lesion of DN is nodular glomerulosclerosis, as described by Kimmelstiel and Wilson.^3^ Renal diseases other than DN can occur in diabetic patients. The absence of retinopathy or neuropathy in patients with evidence of nephropathy may imply that the underlying pathology is unrelated to diabetes.^4^ Other clinical predictors of non-diabetic renal disease (NDRD) in diabetic patients include rapid deterioration of renal function, and microscopic or macroscopic hematuria.^5^ A renal biopsy is not routinely performed in diabetic patients. It is indicated however, in patients where NDRD is suspected.^5^–^7^ The aim of this study was to evaluate the renal biopsies performed on patients with type 2 diabetes for suspicion of NDRD and to correlate the pathology findings with the clinical presentation and laboratory parameters.

## SUBJECTS AND METHODS

All native kidney biopsies performed at Mubarak Al Kabeer Hospital from January 2006 to December 2008 were retrospectively reviewed. Biopsies performed on patients with type 2 diabetes for clinically suspected NDRD were selected and reviewed. Biopsy indications were the presence of Microscopic hematuria, proteinuria without retinopathy or unexplained rapid worsening of renal function. All patients underwent ultrasound-guided renal biopsy using a gauge 16 coaxial quick-core biopsy set. Written consent was obtained from each patient prior to the procedure. The histopathology glass slides were reviewed and the pathology reports were retrieved from the department of pathology computerized filing system. Each kidney biopsy was prepared by cutting paraffin blocks at 3 um sections and staining 2 slides with peroidic acid schiff, 2 slides for hematoxylin and eosin, 1 slide for Jones Methenamine sliver and one slide for trichrome. Immunoperoxidase staining was also performed routinely on all slides for IgG, IgA, IgM and C3. Antibodies were from Dako and titration was performed according to the leaflets with the antibody vials. Electron Microscopy (EM) is not routinely done on all cases in our institution, however, on selected cases EM was performed and the films were retrieved and reviewed along with the EM report.

Patients were grouped by isolated diabetic glomerulosclerosis (DGS) (group I) and by NDRD on a background of DGS (group II). Non of the studied population had isolated NDRD. Patient data including age, gender, duration of diabetes, presence of retinopathy on fundus examination, presence of hypertension (defined as systolic blood pressure above 140 mm Hg, or diastolic blood pressure above 80 mm Hg or intake of antihypertensive medications) were included. Serum creatinine, glomerular filtration rate as estimated by MDRD (modifecation of diet in renal disease study) formula,^8^ presence of hematuria defined as 2 or more red blood cells per high-power field in a centrifuged urine sample prior to biopsy,^9^ and proteinuria using 24 hours urine protein were all considered.

The differences between the two groups were assessed using the χ^2^ test for categorical variables and the nonparametric Mann Whitney U test for continuous variables. Significance was evaluated using a two sided *P*<.05. Statistical analysis was performed using SPSS for windows version 16 (SPSS, Inc, Chicago, IL)

## RESULTS

**Figure 1:**
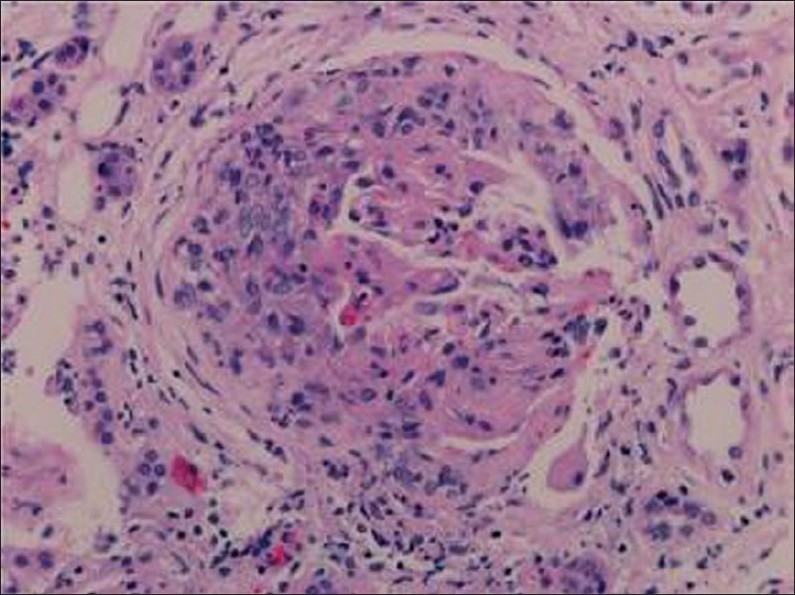
A cellular crescent is present with disruption of the Bowman's space (hematoxylin-eosin stain ×200).

**Figure 2:**
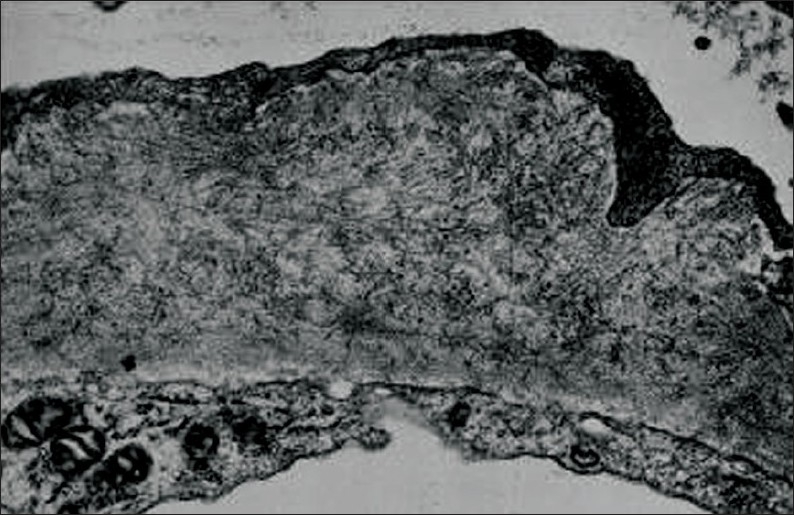
Fibrillary EM; randomly arranged fibrils deposited within the glomerular basement membrane (EM ×80 000).

A total of 312 renal biopsies from native kidneys done during the study period, 31 (9.9%) were performed on patients with type 2 diabetes. Seventeen (54.8%) patients were males. Their mean age was 50.68 (11.29) years. The mean duration of diabetes was 9.33 (3.6) years (Table 1). Indications for renal biopsy were the presence of hematuria in 15 (48.4%) patients, unexplained rapid deterioration of renal function in 10 (32.26%) patients and sudden onset of massive proteinuria without retinopathy in 6 (19.3%) cases. Renal biopsy should that among the studied population 17 (54.8%) cases were found to have isolated diabetic glomerulosclerosis whereas the remaining 14 (45.2%) subjects had nondiabetic renal diseases superimposed on diabetic glomerulosclerosis (Figures 1 and 2).

**Table 1:** Clinical and laboratory data for the studied diabetic patients (n=31).

Patient characteristics	
Age in years mean (SD)	50.68 (11.29)
Male gender (%)	17 (54.8)
Hypertension (%)	17 (54.8)
Retinopathy (%)	14 (45.2)
Duration of diabetes in years mean (SD)	9.33 (3.6)
Creatinine μmol/L mean (SD)	395.23 (299.8)
GFR ml/min/1.73 m^2^ mean (SD)	38.6 (21.0)
Urinary proteins gm/24 hours mean (SD)	3.18 (1.6)
Hematuria (%)	15 (48.4)

Histopathological findings in group II patients showed pauci-immune crescentic glomerulonephritis as the commonest finding seen in three cases (21.4%), acute tubulointerstitial nephritis and hypertensive changes each in 2 cases (14.3%), IgA nephropathy with mesangial IgA deposits in one case (7.1%), immune complex mediated membranoproliferative glomerulonephritis (MPGN) with immune deposits of C3, IgM and IgG in the mesangium and peripheral capillary loops in one case (7.1%), fibrillary glomerulonephritis with mesangial deposits of C3, IgG, IgA and IgM was seen in one patient (7.1%). Other pathological findings are summarized in Table 2. Group I was found to have a significantly higher level of proteinuria than group II (*P*=.003). Retinopathy was significantly higher in group I than in group II (*P*=.008, OR=8.8, CI 1.69-45.76)). No statistically significant difference was detected between the two groups in age at time of biopsy (*P*=.49), duration of diabetes (*P*=.17), gender (*P*=.1), presence of hypertension (*P*=.12), hematuria (*P*=.1), creatinine level (*P*=.86) or creatinine clearance at time of presentation (*P*=.74) (Table 3).

**Table 2:** Histopathological findings in group II (n=14).

Histopathological findings	Number of patients (%)
Crescentic glomerulonephritis	3 (21.4)
Acute tubulointerstitial nephritis	2 (14.3)
Hypertensive changes	2 (14.3)
IgA nephropathy	1 (7.1)
Granulomatous interstitial nephritis	1 (7.1)
Fibrillary glomerulonephritis	1 (7.1)
Primary focal segmental glomerulosclerosis	1 (7.1)
Immune complex mediated membranoproliferative glomerulonephritis	1 (7.1)
Tubular injury secondary to rhabdomyolysis	1 (7.1)
Acute pyelonephritis	1 (7.1)

**Table 3:** Comparison of patients with isolated diabetic glomerulosclerosis (group 1) and patients with non diabetic lesions on top of diabetic glomerulosclerosis (group II) in the demographic, clinical and laboratory characteristics.

Parameter	Group I (DGS)	Group II NDRD	*P* value
Age in years mean (SD)	52.13 (13.2)	49.3 (9.4)	>.05
Male gender (%)	9 (64.3)	8 (47.05)	>.05
Hypertension (%)	8 (50)	9 (60)	>.05
Retinopathy (%)	11 (68.8)	3 (20)	.008
Duration of diabetes in years mean (SD)	10.06 (3.31)	8.27 (3.8)	>.05
Serum creatinine μmol/L mean (SD)	395.56 (335.09)	376.2 (266.58)	>.05
GFR ml/min/1.73 m^2^ mean (SD)	35.21 (17.5)	41.7 (15.02)	>.05
Urinary proteins g/24 hours mean (SD)	4.97 (2.08)	2.72 (1.09)	.003
Hematuria (%)	7 (43.8)	8 (53.3)	>.05

## DISCUSSION

Diabetic nephropathy is one of the most frequent and clinically important complications of diabetes, affecting approximately 40% of patients who have had diabetes for more than 20 years and contributing to a substantial number of patients entering into ESRD programs.^10^ Among patients with type 2 diabetes the prevalence of NDRD varies widely depending on the selection criteria and the populations being studied.^6^,^11^ The frequency of NDRD in diabetics has been reported to be 7 to 44%.^11^

In the present study NDRD was detected in 45.8% of biopsies of diabetic patients. This was in accordance with previous studies where the prevalence of NDRD was found to range from 45% to 57%,^6^,^12^–^14^ but different from other studies where the prevalence of NDRD was around 7-10%.^15^,^16^ This low prevalence could be explained by the different selection criteria for doing renal biopsy in such patients. In the present study no significant difference was found between the two groups in age at the time of biopsy. In contrast, Soni et al^10^ reported that patients with NDRD on the background of DGS were older than those with isolated DGS. The present study did not show a significant difference in the duration of diabetes between the two groups. This was in accordance with Bertani et al^11^ whereas, Soni et al^10^ reported a shorter duration of diabetes with NDRD. Our study was in accordance with the previous study that showed no significant difference in the incidence of associated hypertension between the two groups.^10^ There was also no significant difference between the two studied groups in serum creatinine or creatinine clearance. This was in contrast to the results of Soni et al^10^ and Taft et al^17^ where azotemia was higher in patients with NDRD on top of DGS. Our results were in accordance with previous studies that showed a significantly higher proteinuria in patients with DGS than in patients with NDRD on top of DGS.^6^,^10^ Also, the present study agrees with Pham et al^7^ in showing that hematuria is not a useful predictor of diabetic or non-diabetic renal disease. Our study confirmed the accepted view that one of the important predictors of NDRD is the absence of retinopathy.^10^,^13^,^18^ However, the presence of retinopathy should not rule out the need for renal biopsy, especially if the clinical scenario is atypical. Our study showed that crescentic glomerulonephritis was the commonest NDRD detected in the studied biopsies, whereas Soni et al^10^ reported that acute tubulointerstitial nephritis and postinfectiuos glomerulonephritis were the commonest NDRD. IgA nephropathy was found to be the commonest NDRD in other studies.^19^,^20^

The mechanism implicated in the development of NDRD in diabetic patients remains speculative. The predisposition of DGS to superimposed nephritis has been attributed to enhanced exposure of antigenic cellular components that triggers immune responses. Also, pre-existing glomerular alterations might favor an immune reaction in the subepithelial space.^10^ Lai et al^21^ however, found no difference in the prevalence of NDRD between patients with and without diabetes and that the coexistence of a different glomerulonephritis in the diabetic kidney might be coincidental.

In conclusion, the present study showed that non NDRD was seen in 45.2% of the studied diabetic population and crescentic glomerulonephritis is the commonest NDRD among diabetic patients. A higher level of proteinuria was reported among those with NDRD superimposed on DGS. So, conducting a renal biopsy in diabetic patients is important when the clinical scenario is not typical of diabetic nephropathy as many of the pathological lesions found in our group of patients had the potential for treatment with agents other than the standard angiotensin converting enzyme inhibitors or angiotensin receptor blockers commonly used in typical DGS. Our study has some limitations. It was a retrospective single centre study that included a small number of patients from the same geographical area. A more longitudinal study needs to be done on a larger number of diabetic patients to evaluate the actual prevalence of NDRD in diabetic patients.
